# *Mycobacterium polyniensis* sp.nov, a non-tuberculous mycobacterium clinical isolate from Tahiti, French Polynesia

**DOI:** 10.1016/j.nmni.2025.101691

**Published:** 2026-01-05

**Authors:** M.L. Keita, M. Morsli, M. Drancourt, M. Levy, G. Grine

**Affiliations:** a Aix Marseille Université, IRD, MEPHI, AP-HM, Marseille, France; b IHU Méditerranée Infection, Marseille, France; c VBIC, INSERM U1047, Univ Montpellier, Service de Microbiologie et Hygiène Hospitalière, CHU Nîmes, Nîmes, France; d Laboratoire de Microbiologie, Centre Hospitalier de Polynésie Française, Papeete, French Polynesia

**Keywords:** Polynesia, Mycobacteria novel species, Mycobacteria, *Mycobacterium terrae* complex, *Mycobacterium polyniensis*, Nontuberculous mycobacterium

## Abstract

In this study, we investigated a previously unidentified respiratory tract isolate, CSUR_Q5927, collected in Tahiti, French Polynesia. Through a comprehensive polyphasic approach combining phenotypic traits with whole genome sequencing, we sought to elucidate the identity and characteristics of this isolate. After cultivating colonies in Middlebrook 7H10 at 37 °C for nine days, we observed acid-fast bacilli under microscopic examination and Ziehl-Neelsen staining. Additionally, electron microscopy revealed the presence of unsporulated bacilli, approximately 1.27 μm ± 0.26 μm in size, displaying morphology consistent with that of a mycobacterium. Matrix-assisted laser desorption/ionization mass spectrometry provided an identification score of 1.42, indicating a low confidence level. However, clustering analysis grouped isolate CSUR_Q5927 within the *Mycobacterium terrae* complex. Whole genome sequencing unveiled a CG-content of 68.5 % and a coding ratio of 93.6 %, comprising 4337 genes encoding proteins, 55 tRNA genes, and two rRNA. Further comparison with reference strain *Mycobacterium terrae* NCTC 10856 using DNA-DNA hybridisation values revealed a similarity of 35.8 %, confirming the presence of a new species within the *M. terrae* complex. In vitro susceptibility testing demonstrated the isolate's susceptibility to eight antimicrobials and resistance to five others. After meticulously conducting appropriate negative controls and analysing all the data, we propose the name *Mycobacterium polyniensis* for this novel *M. terrae* species, with isolate CSUR_Q5927 serving as the prototype strain. This discovery expands our understanding of the microbial diversity in the Polynesian region and highlights the importance of combining advanced techniques to accurately characterise and classify previously unknown mycobacteria species.

## 1 Introduction

Nontuberculous mycobacteria (NTM) are emerging as opportunistic pathogens worldwide [1,2]**.** They comprise several species mainly grouped into the *Mycobacterium abscessus* [[3], [4], [5]]**,** *Mycobacterium avium* [6,7]**,** *Mycobacterium terrae* [8] and *Mycobacterium fortuitum* [9] complexes. Hundreds of reports from the five continents address both environmental and clinical NTM. However, NTM continue to be overlooked [10] in some remote areas [11]**,** obscuring the overall understanding of these organisms as well compromising the diagnosis and medical care of exposed patients.

French Polynesia is one of the 22 Pacific Island countries and territories of the Oceania region, remotely located in the South Pacific [2,12]. French Polynesia comprises five archipelagos of 118 islands which are home to 274 000 inhabitants [2,13]. Little is known regarding NTM in this wide yet remote region due to the lack of laboratory facilities, with the exception of French Polynesia which does have medical facilities [12]. Diagnosis of mycobacterial infections most often relies on microscopic observation of acid-fast bacilli in the clinical samples and culture is not routinely performed [[14], [15], [16], [17]].

While new *Mycobacterium tuberculosis* genotypes including the query *Mycobacterium canettii* [13,13,[18], [19], [20]] and several NTMs have been reported from patients living in one of the 67 inhabited islands of French Polynesia [2,21], a few novel NTMs of clinical interest have been reported from French Polynesia over the eight past years [2], including *Mycobacterium massilipolynesiensis* close to *Mycobacterium phlei* [22] and *Mycobacterium mephinesia* in the *Mycobacterium terrae* (*M. terrae*) complex [8].

In this work, we extended the repertoire of described NTM species in French Polynesia by describing a new *Mycobacterium* species named *Mycobacterium polyniensis* sp.nov, isolated from pulmonary secretions collected in Tahiti, French Polynesia.

## 2 Materials and methods

**Phenotypic characterisation.** Strain CSUR_Q5927 was isolated in the microbiology laboratory in Papeete, Tahiti, from bronchial secretions obtained by fibreoptic bronchoscopy from a patient in his forties, admitted with pseudonodular lesions of the upper lobe of the right lung. Growth of the CSUR_Q5927 strain was evaluated in different culture media including MGIT (Mycobacterial Growth Indicator Tube; Becton Dickinson, Strasbourg, France), Middlebrook 7H10, Coletsos (Biorad, Marnes-la-Coquette, France), and Löwenstein-Jensen (Becton Dickinson). The study of the temperature and the optimal atmospheric conditions for growth consisted in inoculating a pure culture of CSUR_Q5927 strain on three Middlebrook 7H10 medium tubes placed in zip-lock bags and incubated at 8 °C, 30 °C, 37 °C, 45 °C and 56 °C under aerobic, anaerobic, and micro-aerobic conditions. Subcultures were observed after 24 hours, 48 hours, and 72 hours and then daily for 14 days. CSUR_Q5927 strain morphology was observed using an optical microscope (Leica DM 2500, Leica, Wetzlar, Germany) and photographed with a Nikon Digital Sight DS-U1 camera (Nikon, Tokyo, Japan) at 100X after Gram and Ziehl-Neelsen staining. For electron microscopy, colonies collected from pure culture on Middlebrook 7H10 agar medium supplemented with 10 % OADC (Becton Dickinson) were fixed in 2.5 % glutaraldehyde for 1 h and then cytocentrifuged on glass slides and sputtered with a 10 μm thick platinum layer. Micrographs were recorded on SU5000 SEM, using the backscatter electron detector in high vacuum mode (Hitachi High-Technologies, Tokyo, Japan). Bacterial length and width were measured for 50 mycobacteria after acquisition using Fiji's ImageJ software [23]. The oxidase test was performed by applying the oxidase solution (Becton Dickinson) to a mass of colonies placed on Whatman filter paper (Becton Dickinson). The positivity of the test was confirmed by the emergence of a purple colour. For the detection of catalase activity, one colony was mixed with a drop of ID.Color Catalase hydrogen peroxide (bioMérieux, Marcy l’Etoile, France) deposited on a microscopic slide. A positive reaction was characterised by the instantaneous appearance of a cloudy colour. Enzymatic profiling was carried out using API ZYM and API CH50 strips in accordance with the supplier's recommendations (bioMérieux). CSUR_Q5927 strain sporulation capacity was evaluated by inoculating 100 μL of mycobacterial suspension on Middlebrook 7H10 agar after heating at 80 °C for 20 min, incubated at 37 °C for 14 days and observed daily.

**Antibiotic susceptibility testing**. The antibiotic susceptibility test was performed by inoculating a one MacFarland suspension on Middlebrook 7H10 agar supplemented with 10 % OADC (Becton Dickinson) and incubated at 37 °C for 24 hours [24] (https://www.ncbi.nlm.nih.gov/books/NBK544374/). An Etest antibiotic strip (bioMérieux) was applied to each agar plate and maintained at 37 °C for two weeks followed by daily observation from the fifth day of incubation [24,25]. Antibiotic susceptibility was assessed by extrapolating the measure of the inhibition zone into a minimum inhibitory concentration (MIC) plotted on the strips. The antibiotics included aminoglycosides (amikacin), rifamycins (rifampicin), antituberculosis drugs (ethambutol, isoniazid), trimethoprim, sulphonamides (sulfamethoxazole, trimethoprim-sulfamethoxazole combination), fluoroquinolones (moxifloxacin, levofloxacin, ciprofloxacin), oxazolidinones (linezolid), beta-lactams (imipenem), phenicols (chloramphenicol), and tetracyclines (doxycycline).

**Mass spectrometry profiling**. The peptide profile was obtained using matrix-assisted laser desorption/ionization mass spectrometry (MALDI-TOF/MS) as previously described [26]**.** Briefly, the α-cyano hydroxycinnamic acid (CHCA) matrix solution was applied to two positive control spots consisting in a deposit of 1.5 μL of a solution of Bacterial Test Standard (Bruker Daltonics, Bremen, Germany), a commercial protein extract of *Escherichia coli*, as well as four sample spots. Two negative controls spots were also added, consisting only in two 1.5μL matrix solution deposits. The spots were analysed with a Microflex device (Bruker Daltonics, Bremen, Germany) with a mass range between 2000 Da and 20 000 Da in linear positive mode. Spectra obtained were then identified using the MBT Compass biotyping software v4.1 (Bruker Daltonics). The positive controls were confirmed to be *E. coli* and no identifications were obtained for negative controls.

**Whole genome sequencing.** After ten colonies were resuspended in 200 μL of phosphate-buffered saline (PBS), DNA was extracted using the EZ1 DNA Tissue kit (Qiagen, Courtaboeuf, France) and eluted in a 50-μL volume. Whole genome sequence (WGS) was then generated by using the Illumina pair-end sequencing (Illumina, San Diego, California, USA) platform, followed by the Nextera-XT V2 library preparation protocol and then sequenced on an Illumina MiSeq, as previously described At the same time, they were sequenced using Oxford Nanopore single-long reads library preparation then sequenced for 24 hours on a MinION instrument (Oxford Nanopore Technologies, Oxford, UK)**.** Both Illumina and Nanopore data were quality controlled using FastQC software (Version 0.73) on the European Galaxy online platform (https://usegalaxy.eu/). The Illumina and Nanopore reads were concatenated and *de novo* assembled using Unicycler software version (Version 0.4.8.0). The generated contigs were blasted against NCBI GenBank database and a phylogenetic tree based on WGS was generated using the Orthologous Average Nucleotide Identity Tool (OAT) software version (0.93.10) with standard parameters [27] (Fig. 2). The strain CSUR_Q5927 genome was annotated on DDBJ Fast Annotation and Submission Tool online platform (https://dfast.ddbj.nig.ac.jp/). The phylogenetic analysis based on 16S rRNA gene sequence with the ten hit-blasts was performed using Neighbour-Joining BioNJ algorithm standard parameters on MEGA software (version 7.0.26). Genome representation was performed using the Proksee platform with standard parameters (version 1.0.0). (https://proksee.ca/). Blastp was used against the Clusters of Orthologous Groups (COG) database to find putative protein functions for strain CSUR_Q5927, *Mycobacterium sinense* and *Mycobacterium terrae (M. terrae).*

## 3 Results

**Fig. 1:**
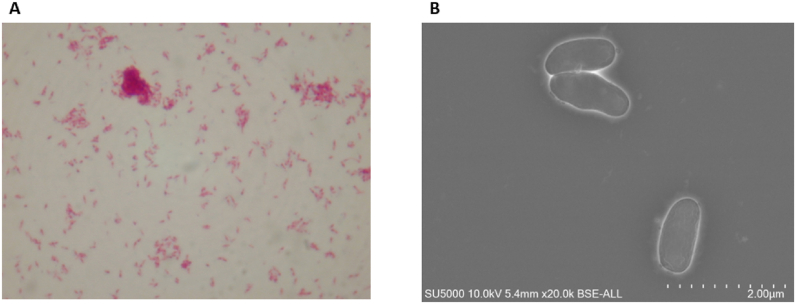
Phenotypic traits of *Mycobacterium polyniensis* sp. nov., Q5927 strain. **(A)** Ziehl-Neelsen coloration on the isolated strains **(B)** Morphological features of isolated strain using electronic microscopy TM4000.

**Phenotypic characteristics of CSUR_Q5927 strain.** Strain CSUR_Q5927 presented as pink-stained, acid-alcohol-fast rods measuring 1.27 μm ± 0.26 μm × 0.542 μm ± 0.06 μm (Fig. 1). Growth occurred in MGIT tubes after 16 days and on Middlebrook 7H10 after nine days, forming non-chromogenic, smooth colonies under both aerobic and microaerophilic conditions. MALDI-TOF-MS produced reproducible spectra with no database matches, though dendrogram analysis clustered CSUR_Q5927 with *Mycobacterium kumamotonense* (Fig. 1). The strain was catalase-positive, oxidase-negative, and showed positive reactions for D-lactose, D-saccharose, potassium 5-ketogluconate, and multiple enzymes (phosphatase alkaline, esterases, arylamidases, β-galactosidase). Antimicrobial susceptibility testing revealed sensitivity to ethambutol (MIC 3 mg/L), doxycycline (0.38 mg/L), levofloxacin (0.25 mg/L), and amikacin (0.125 mg/L), with resistance to linezolid, isoniazid, and trimethoprim-sulfamethoxazole.

**Fig. 2:**
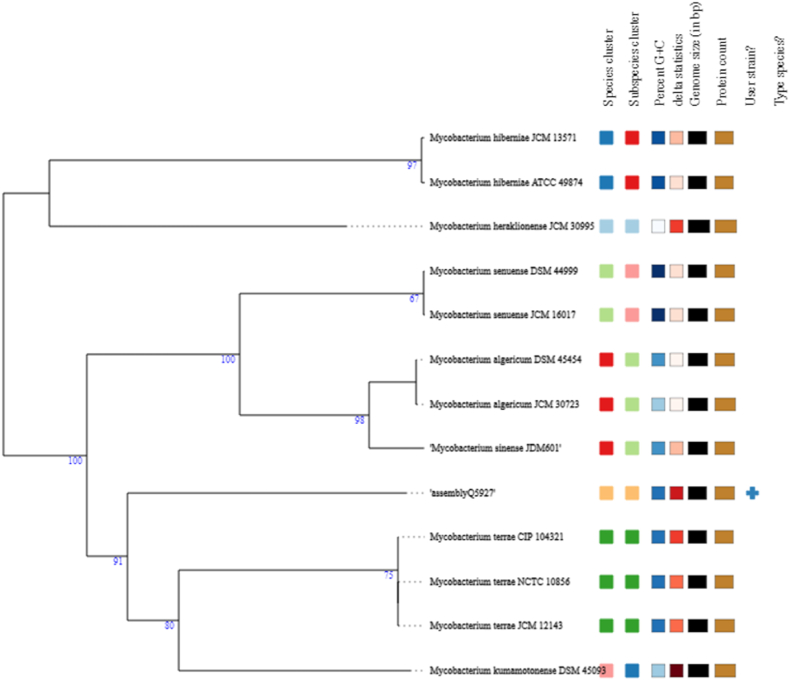
Phylogenetic tree based on whole genome analysis of *Mycobacterium polyniensis* Q5927 strain generated directly on the TYGS online platform. The *M. polyniensis* shared 91 % sequence similarity with *Mycobacterium kumamotonense* and *Mycobacterium terrae*.

### 3.1 Genomic characterisation

**Fig. 3:**
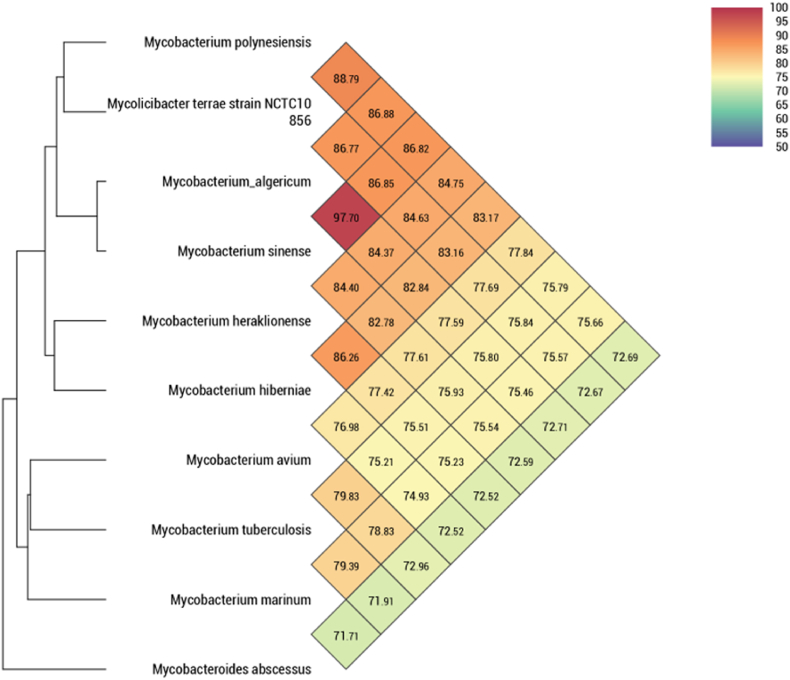
Genome features of the *M. polyniensis* genome. Whole genome sequence-based phylogeny. *Mycobacterium polynesiensis* shared 88.79 % genome similarity with *Mycobacterium terrae*, 86.88 % similarity with *Mycobacterium algericum*, 86.82 % with *Mycobacterium sinense*, 84.64 % with *Mycobacterium heraklionense*, and 83.17 % similarity with *Mycobacterium hiberniae*. The other Mycobacterial species including *Mycobacterium tuberculosis*, *Mycobacterium avium*, *Mycobacterium marinum*, and *Mycobacterium abscessus* have less than 78 % sequence similarity with the new isolated *Mycobacterium polynesiensis. The heatmap was generated with OrthoANI values calculated from the software* [27].

**Genome assembly and annotation.** Whole genome sequencing generated 4 509 467 bp across five contigs with 68.5 % GC content. The genome encoded 4337 protein-coding genes, 55 tRNAs, and two rRNAs (93.6 % coding density; Fig. 2, Table 1). Comparative genomics showed highest similarity to *M. terrae* NCTC 10856 (88.79 %), followed by *M. algericum* (86.88 %) and *M. sinense* (86.82 %), with <78 % similarity to other mycobacterial species including *M. tuberculosis* and *M. avium* (Fig. 3).

**Table 1:** Genome annotation of *Mycobacterium polynesiensis*.

Genome statistics	
**Total length (bp)**	4 509 467
**No. of sequences**	5
**GC content (%)**	68.5 %
**N50**	3 894 844
**Gap ratio (%)**	0.0 %
**No. of CDSs**	4357
**No. of rRNA**	2
**No. of tRNA**	55
**No. of CRISPRS**	0
**Coding ratio (%)**	93.6 %
**dDDH**	35.8

### 3.2 Mass spectrometry profiling

**Fig. 4:**
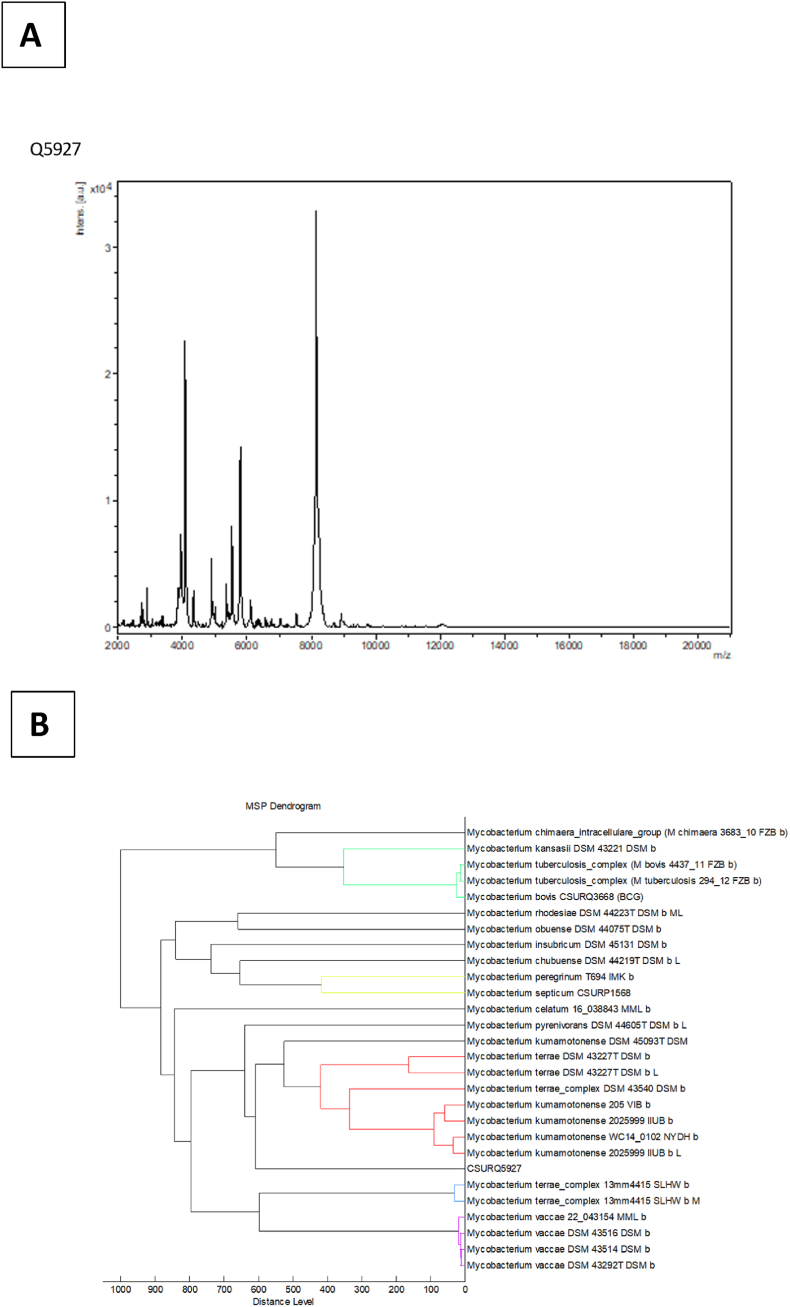
**A.** Maldi-ToF spectra of the CSUR Q5927 isolate. **B.** Dendrogram of the CSUR Q5927 strain based on MALDI-TOF spectra similarity with best matching hints in databases available, generated with MBT Compass Explorer (Bruker). MSP generated on spectra from CSUR Q5927 strain, designated as “CSURQ5927”, clustered with a distance of 609 arbitrary units with a group constituted from species *Mycobacterium terrae* and *Mycobacterium kumamotonense*.

Four high-quality spectra (S/N > 3) yielded a best match to *M. kumamotonense* (score 1.42, below the 2.00 identification threshold; Fig. 4A). Dendrogram analysis positioned the CSUR_Q5927 main spectrum profile within the *M. terrae* complex cluster (distance: 609 arbitrary units) while remaining distinct from all known complex species in reference databases (Fig. 4B).

## 4 Discussion

We report on the polyphasic characterisation of a respiratory tract isolate CSUR_Q5927 leading to the description of a new species in the *M. terrae* complex, with all reported data being validated by the negativity of negative controls, for which we propose the name *M. polyniensis* (*M. polyniensis*) sp. nov (polyne.n'us, N.L. gen. n. polyniensis, of the Polynesia, strain isolated from a patient in Polynesia), from the name of the island of Polynesia (France) where it was isolated. The CSUR_Q5927 strain exhibited a unique peptidic profile with no match in the CSUR MALDIT-TOF-MS database comprising 1083 entries for 152 different Mycobacterium species yet indicating that Q5927 was representatives of a *M. terrae* complex taxon, as validated by controls. Furthermore, CSUR_Q5927 exhibited a unique biochemical profile within the members of this *M. terrae* complex. DDH values derived from Q5927 also agreed on a new species within this complex. The *M. terrae* complex includes species of slow-growing mycobacteria of environmental origin, responsible for various infections involving the bones, joints and lungs [28]**.** Its taxonomic diversity previously composed of *M. terrae*, *M. nonchromogenicum* and *M. triviale* has been extended to many species including *M. kumamotonense*, *M. senuense*, *M. paraterrae*, *Mycobacterium JDM601*, *M. engbaekii*, *M. longobardum*, *M. heraklionense*, *M. virginiense* and *M. arupense,* thanks to the advent of new genomic capabilities such as next generation sequencing (NGS) [28,29]**.**

Enzymatic activity due to catalase and β-galactosidase is most active in members of the *M. terrae* complex [30]**.** During our experiments, these enzymes were demonstrated in *M. polynienesis*.

Pulmonary infections involving the *M. terrae* complex are rare, although they have been regularly reported since their first notification in 1983 [31,32]**.** Durant [29] reported lung infection with *M. terrae* in a 65-year-old patient after bronchoscopy and bronchoalveolar lavage. Thirty years earlier, characterised by the insufficiency of molecular microbiology identification techniques in laboratories, Krisher had described an infection due to the *M. terrae* complex in a 29-year-old patient [31]**.** Analysis of the antimycobacterial specific antibiogram shows that the *M. polyniensis* strain is sensitive to ethambutol and rifampicin. Indeed, the sensitivity of strains belonging to the *M. terrae* complex to ethambutol had previously been reported by various studies [33,34]. Regarding the resistance of *M. polyniensis* to linezolid, trimethoprim-sulfamethaxazol and trimethoprim, our results agree with those of Beam relating to *Mycobacterium arupense* [33].

Diagnosis and control of NTM infections require their isolation and culture. Concomitantly with phenotypic and biochemical identification techniques and evaluation of antimicrobial susceptibility, genomic analyses are seeing considerable success today for the isolation and description of Mycobacteria, including NTMs**.**

## CRediT authorship contribution statement

**M.L. Keita:** Investigation, Methodology, Writing – original draft, Writing – review & editing. **M. Morsli:** Conceptualization, Data curation, Investigation, Methodology. **M. Drancourt:** Conceptualization, Data curation, Supervision, Validation, Writing – original draft. **M. Levy:** Conceptualization, Resources. **G. Grine:** Conceptualization, Data curation, Formal analysis, Investigation, Methodology, Supervision, Validation, Visualization, Writing – original draft, Writing – review & editing.

## Declaration of competing interest

All the authors declare that there are no conflicts of interest.
